# Variant BCR-ABL1 fusion genes in adult Philadelphia chromosome-positive B-cell acute lymphoblastic leukemia

**DOI:** 10.17179/excli2017-793

**Published:** 2017-10-18

**Authors:** Stephen E. Langabeer

**Affiliations:** 1 Cancer Molecular Diagnostics, St. James's Hospital, Dublin, Ireland

## ⁯

Dear Editor,

Acute lymphoblastic leukemia (ALL) is the manifestation of malignant transformation and subsequent proliferation of either B- or T-lymphoid progenitor cells than manifests predominantly in the bone marrow. ALL is more frequent in children in whom long term survival has vastly improved in recent years, however, in adults this malignancy remains clinically challenging (Terwilliger and Abdul-Hay, 2017[21]). The recent World Health Organization classification of acute leukemias considers subdivision of ALL types on the basis of cytogenetic and molecular abnormalities among which is B-cell lymphoblastic leukemia/lymphoma with the t(9;22)(q34;q11.2)/Philadelphia chromosome and *BCR-ABL1 *rearrangement (Ph+ ALL) (Arber et al., 2016[1]). Ph+ ALL is uncommon in childhood but increases in incidence with advancing age of presentation. The introduction of tyrosine kinase inhibitors into existing and new treatment regimens has improved the outlook for many adult Ph+ ALL patients resulting in the increased ability to proceed to hematopoietic allogeneic stem cell transplantation (Ronson et al., 2017[17]). Most Ph+ ALL treatment algorithms now incorporate some measure of minimal residual disease (MRD) response into risk stratification which may be achieved through a number of laboratory approaches. These approaches need to be sensitive, fast, with a requirement for standardization (van Dongen et al., 2015[23]).

**Figure 1:**
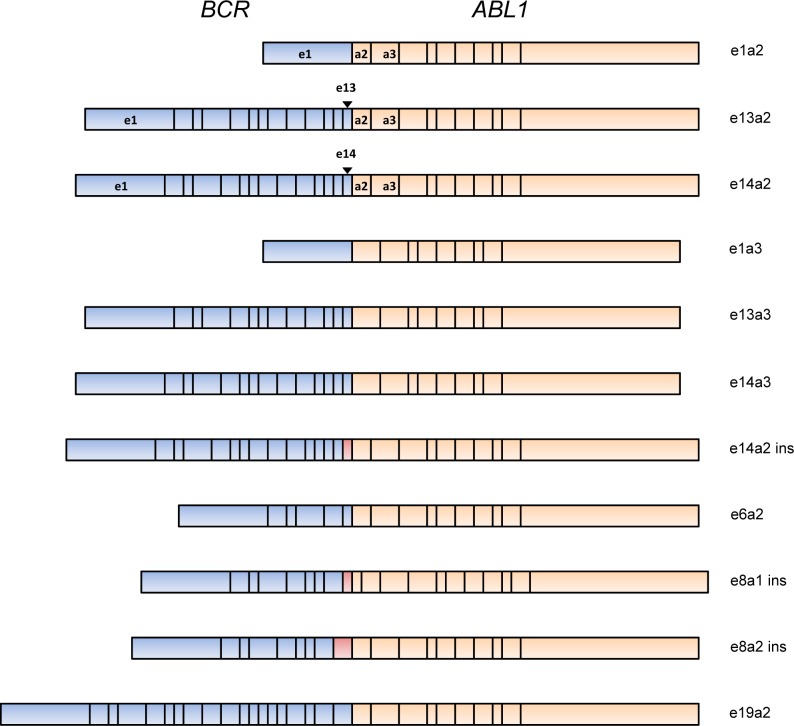
Exonic structure of the variant *BCR-ABL1* transcript types reported in adult Ph+ ALL. ins: inserted sequence

Monitoring *BCR-ABL1 *transcripts for MRD by real-time quantitative reverse transcription polymerase chain reaction (RT-qPCR) is now an essential component in the management of Ph+ chronic myeloid leukemia and this approach may also be applied to Ph+ ALL patients as a means of assessing MRD and therefore therapeutic efficacy. While the vast majority of Ph+ ALL patients express either the common e1a2, or less frequent e13a2 or e14a2 *BCR-ABL1* fusion transcripts (Figure 1(Fig. 1)), a minority harbor variants, usually as a consequence of alternative splicing of either *BCR* or *ABL1* exons. Characterization of the exact *BCR-ABL1* fusion gene at diagnosis is therefore critical for design and selection of primers and probes for RT-qPCR analysis. Summarized within are the variant *BCR-ABL1* fusions that have been reported in Ph+ ALL to date (Table 1(Tab. 1); References in Table 1: Soekarman et al., 1990[19]; Iwata et al., 1994[10]; Wilson et al., 2000[24]; Burmeister et al., 2007[3]; Fujisawa et al., 2008[8]; Langabeer et al., 2011[14]; Chen et al., 2013[5]; Shin et al., 2015[18]; Sonu et al., 2015[20]; López-Andrade et al., 2016[15]; Burmeister et al., 2007[3]; Zhang et al., 2016[25]; Kurita et al., 2016[13]; Hirota et al., 2000[9]; Burmeister et al., 2007[3]; Deshpande et al., 2016[7]; McCarron et al., 2011[16]; Kim et al., 2012[12]; Jeon et al., 2011[11]) and that result in the presence or absence of the encoded functional domains of the oncogenic *BCR-ABL1* protein contributing to altered cellular adhesion, enhanced proliferation, inhibition of apoptosis and increased genomic instability of Ph+ ALL (Figure 1(Fig. 1)).

Detection of the variant *BCR-ABL1* fusion genes should be considered when molecular and cytogenetic findings are discordant and can be achieved by a number of different RT-PCR strategies (Cross et al., 1994[6]; van Dongen et al., 1999[22]; Chasseriau et al., 2004[4]; Burmeister and Reinhardt, 2008[2]) with confirmation necessary by sequencing of atypical PCR products. As these variants are present in only a minority of Ph+ ALL cases, their influence on genotype and impact on outcome remain unknown.

## Conflict of interest

The author declares no conflict of interest.

## Figures and Tables

**Table 1:**
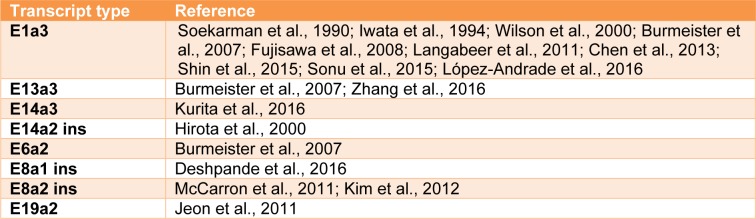
Variant *BCR-ABL1* transcript types reported in adult Ph+ ALL
